# Modulatory Effect of *Trypanosoma cruzi* Infective Stages in Different Dendritic Cell Populations *in vitro*

**DOI:** 10.3389/fcimb.2020.00020

**Published:** 2020-02-27

**Authors:** Brenda Celeste Gutierrez, Estela Lammel, Marcel Ivan Ramirez, Stella Maris González-Cappa, Carolina Verónica Poncini

**Affiliations:** ^1^Laboratório de Inmunología Celular e Inmunopatología de Infecciones, Instituto de Investigaciones en Microbiología y Parasitología Médica (MPaM) UBA-CONICET, Buenos Aires, Argentina; ^2^Instituto Oswaldo Cruz, FIOCRUZ, Rio de Janeiro, Brazil; ^3^Departamento de Bioquímica, Universidade Federal Do Paraná, Curitiba, Brazil; ^4^Departamento de Microbiología, Parasitología e Inmunología, Facultad de Medicina, Universidad de Buenos Aires, Buenos Aires, Argentina

**Keywords:** *Trypanosoma cruzi*, dendritic cells, cell activation, cytokines, T cells

## Abstract

*Trypanosoma cruzi* is a protozoan parasite that infects at least 7 million persons in the world (OMS, 2019). In endemic areas, infection normally occurs by vectorial transmission; however, outside, it normally happens by blood and includes congenital transmission. The persistence of *T. cruzi* during infection suggests the presence of immune evasion mechanisms and the modulation of the anti-parasite response to a profile incapable of eradicating the parasite. Dendritic cells (DCs) are a heterogeneous population of antigen-presenting cells (APCs) that patrol tissues with a key role in mediating the interface between the innate and adaptive immune response. Previous results from our lab and other groups have demonstrated that *T. cruzi* modulates the functional properties of DCs, *in vitro* and *in vivo*. During vectorial transmission, metacyclic (m) trypomastigotes (Tps) eliminated along with the insect feces reach the mucous membranes or injured skin. When transmission occurs by the hematic route, the parasite stage involved in the infection is the circulating or blood (b) Tp. Here, we studied *in vitro* the effect of both infective mTp and bTp in two different populations of DCs, bone marrow–derived DCs (BMDCs) and XS106, a cell line derived from epidermal DCs. Results demonstrated that the interaction of both Tps imparts a different effect in the functionality of these two populations of DCs, suggesting that the stage of *T. cruzi* and DC maturation status could define the immune response from the beginning of the ingress of the parasite, conditioning the course of the infection.

## Introduction

*Trypanosoma cruzi*, the etiological agent of Chagas disease, is a protozoan parasite that affects 7 million people in the world (OMS, 2019).

The parasite life cycle includes at least three particular morphological stages. In the insect vector, there is the presence of multiplicative epimastigotes and infective metacyclic trypomastigotes (mTp), released with feces. In the mammal host, two stages are present, the intracellular multiplicative amastigotes and bloodstream trypomastigotes (bTp), involved in the parasite dissemination to tissues. While vectorial and oral transmission involves mTp entry via injured skin or mucous membranes, parasite dissemination in the mammalian host occurs by bTp, also responsible for congenital and sanguineous transmission even in non-endemic regions (de Souza et al., 2014; OMS, 2019).

A limited number of studies compare the immune response against different *T. cruzi* infective stages.

In a comparative study between mTp and bTp, Dias et al. (2013) have demonstrated that the infection by intraperitoneal or oral inoculation of bTp is more virulent than with mTp. Specifically, animals infected with bTp presented higher parasitemia and mortality in comparison to infection with mTp (Dias et al., 2013).

Dendritic cells (DCs) are professional antigen-presenting cells (APCs) with a central role in the development of immunity against infections. In steady state, DCs patrol tissues in order to maintain immune homeostasis. Under certain conditions such as inflammation or infection, these cells can be activated after recognizing danger signals. This process includes uptake and processing of antigens for presentation in addition to DC migration to draining lymph nodes (LNs). DCs are responsible for T-cell priming, the activation of secondary immune responses, or the induction of tolerogenic programs (Merad et al., 2013).

Previous studies from our group demonstrated that bTp from the virulent strain RA (Gonzalez Cappa et al., 1981) negatively regulates bone marrow–derived DC (BMDC) activation *in vitro*. The parasite promotes the production of anti-inflammatory cytokines and diminishes the T-cell stimulatory capacity of DCs (Poncini et al., 2008). In addition, our results demonstrate that both live bTp and heat-killed Tp, but not fixed Tp, condition DCs to a tolerogenic profile, suggesting that Tp–DC surface interaction and not infection triggers the DCs polarization. In fact, we confirmed that early in time, Tp–DC interaction induces ERK phosphorylation and IL-10 upregulation via a TLR-4–dependent pathway (Poncini et al., 2010).

In a previous report, da Costa et al. (2014) have shown that Tp from strains with variable virulence differentially modulates DC activation *in vitro*. *In vivo*, it was demonstrated that during infection, splenic DCs and APCs display low expression of MHCII and/or costimulatory molecules and impaired function (Alba Soto et al., 2003; Planelles et al., 2003), and more recently, that DCs with tolerogenic properties promote Treg cell induction and parasite persistence (Poncini et al., 2015). The fact that the parasite successfully infects the mammalian host via different portals of entry and by different infective stages highlights the importance the model of infection acquires during experimentation. As a result, a detailed characterization of the interaction of the parasite with cells involved in the first line of defense during primoinfection was needed (Poncini and González-Cappa, 2017).

This work comparatively describes the effect of two different infective stages of a virulent strain of *T. cruzi* (RA) in DCs from different origins. By studying the interaction of bTp and mTp with BMDCs and XS106, a cell line derived from epidemic DCs (Mohan et al., 2005), we found an interesting approach to understand and hypothesize about the different responses that may be occurring at the parasite entry site.

## Materials and Methods

### Animals

C3H/HeN, C57BL/6, and CF1 mice were maintained in the animal facilities of IMPaM UBA-CONICET, Facultad de Medicina, Universidad de Buenos Aires, and bred under a sanitary barrier in specific-pathogen-free conditions (Poncini et al., 2015).

All experiments were performed according to protocol CD N° 04/2015 approved by the University of Buenos Aires's Institutional Committee for the Care and Use of Laboratory Animals (CICUAL) in accordance with the Council for International Organizations of Medical Sciences (CIOMS) and International Council for Laboratory Animal Science (ICLAS) and the international ethical guidelines for biomedical research involving animals.

### Parasites

RA *T. cruzi* bTps were maintained in CF1 mice and obtained from whole blood at the peak of parasitemia (7 days post-infection) by differential centrifugation or by density gradient, using Histopaque-1083 (Sigma-Aldrich) as previously reported (Poncini et al., 2008). Parasites were obtained from the supernatant by centrifugation (10,000 g, 30 min, and 20°C) and resuspended in fresh Iscove's modified Dulbecco's medium (IMDM, Sigma-Aldrich). Epimastigotes were cultured *in vitro* in liver infusion tryptose (LIT) medium at 27°C to the exponential phase of growth and centrifuged at 3,000 g for 15 min at 10°C. The flasks had a liquid depth not exceeding 10 mm and were incubated without agitation for different amounts of time according to the experimental schedule (Isola et al., 1986). mTps were obtained as described by Cestari et al. (2012) with some adjustments. Briefly, after *in vitro* culture of 10 × 10^7^ epimastigotes in 10% fetal bovine serum (FBS) LIT plus Grace's insect medium (MERC) and incubation at 27°C in tightly closed culture flasks, parasites were purified in a DEAE column equilibrated with PBS-glucose (20%) at pH 8.2. Purity was analyzed by microscopic examination.

### BMDCs and XS106 Cell Line Cultures

Bone marrow from C3H/HeN mice was cultured for 7 days as previously described (Poncini et al., 2008). Briefly, bone marrow was flushed from femurs and tibias by syringe and 25-gauge needles with IMDM supplemented with 10% FBS, 100 U/ml penicillin, 100 mg/ml streptomycin, and 50 μM 2-mercaptoethanol (referred to below as 10-IMDM). The tissue was mechanically disaggregated, and DCs were obtained by culturing BM cells, supplemented with 20% supernatant from a GM-CSF–expressing cell line (J558 GM-CSF) at 37°C and 5% CO2. Then, at days 2 and 5, fresh medium was added to the cultures. At day 7, cells displayed a myeloid phenotype (>95 % CD11b) and were highly enriched in DCs (>70 % CD11c).

XS106, a long-term DC line established from newborn epidermis of A/J mice, was kindly provided by Dr. Takashima (University of Toledo, MTA M2014-25). They express a mature phenotype as described by Mohan et al. (2005).

Then, cells were harvested, washed, and plated (1 × 10^6^ cells/ml) in 24-well plates (Nunc, NY, USA) and cultured in conditioned medium (Mohan et al., 2005) with or without 10 μg/ml LPS (*Escherichia coli* O26:B6, Sigma-Aldrich), and/or b/mTp (cell-to-parasite ratio 1:1, 1:4, or 1:10, depending on the experiment) overnight (ON).

### BMDCs and XS106 Cell Culture, Stimulation, and Infection

As described above, cells were cultured in 24-well plates (Nunc, NY, USA) in medium with bTp or mTp (cell-to-parasite ratio 1:1, 1:4, or 1:10, depending on the experiment) ON at 37°C and 5% CO_2_. Then, cells were washed to eliminate parasites in suspension and cultured in fresh medium for 4 or 7 more days. Then, samples were processed by cytospin and cell imprints stained with Giemsa. For immunofluorescence microscopy, cells were washed and stained with anti-mouse CD11c PE-conjugated mAb (BD PharMingen), attached to positively charged glass slides (Fisherbrand, Pittsburgh, PA), fixed in methanol, and stained with anti-parasite rabbit serum or appropriate controls and FITC-conjugated secondary Ab (Sigma-Aldrich).

The percentages of infected cells at the different cell–Tp ratios were defined after microscopic examination (400 × or 1,000 × magnification) by quantifying the relative number of cells with amastigotes in 15 random fields counted per treatment per sample.

### Mixed Lymphocyte Reaction

To characterize APC capacity to induce lymphoproliferation, BMDCs or XS106 cells were cocultured with lymphocytes to test alloresponse as previously reported (Poncini et al., 2008). Briefly, DCs were cultured with different stimuli (medium, LPS, bTp/mTp) for 24 h, harvested, washed, irradiated (30 Gy), and plated with single-cell suspension enriched in T cells prepared from LNs of 8-week-old male C57BL/6 mice after CD3^+^ T-cell purification by positive selection (MiniMACS separation; Miltenyi Biotec). Briefly, T cells (CD3^+^) were purified from LN by using anti-mouse biotin-conjugated CD3 mAb (145-2C11; BD Biosciences) and streptavidin-conjugated microbeads for magnetic positive selection (MiniMACS). Purity was checked by flow cytometry and found to be around 90% of CD3^+^ cells, as previously reported (Poncini et al., 2015). Cells were plated at a 1:10 APC–LN cell ratio, using 1 × 10^5^ LN cells/well, and cultured at 37°C and 5% CO2 in 10-RPMI 1640 medium (Gibco, NY, USA) supplemented with 2 mM L-glutamine, 100 U/ml penicillin, 100 mg/ml streptomycin, and 50 μM 2-mercaptoethanol. Mixed lymphocyte reaction (MLR) assay was performed in 96-well microplates (Nunc) and cultured for 3 days. Proliferation was quantified by Ki-67 (REA183, Miltenyi Biotec) detection by flow cytometry according to the manufacturer's protocol.

### Flow Cytometry

Cells (1 × 10^6^) were washed in ice-cold PBS supplemented with 1% bovine serum albumin and 0.1% NaN_3_, and incubated for 30 min at 4°C with a previously optimized amount of one or more of the following anti-mouse mAbs conjugated to different fluorophores: CD11b-PE (M1/70), CD11c-PE (HL3), I-Ak-FITC (11-5.2), CD40-Biot (3/23), CD80-Biot (16-10A1), and CD86-Biot (GL-1). Finally, cells were fixed with 1% paraformaldehyde. All mAbs and second reagents were purchased from PharMingen or Miltenyi Biotec. Samples were acquired on FACSCalibur (Becton Dickinson), and data were analyzed with FlowJo 7.6.2 software.

### Enzyme Linked Immuno Assay

Cell culture supernatants were collected and stored at −80°C until used. Mouse IL-10 and TNF-α were detected by enzyme linked immune assay (ELISA) (R&D Systems) according to the manufacturer's protocol.

### Statistical Analysis

Two groups were compared with unpaired Student's *t*-test. ANOVA and Bonferroni's or Dunnett's multiple comparison tests were performed in order to analyze statistical significance. All analyses were carried out with GraphPad Prism 4 software for Windows. A *p* < 0.05 value was assumed as significant.

## Results

### XS106 Cell Line Phenotypic Characterization

**Figure 1:**
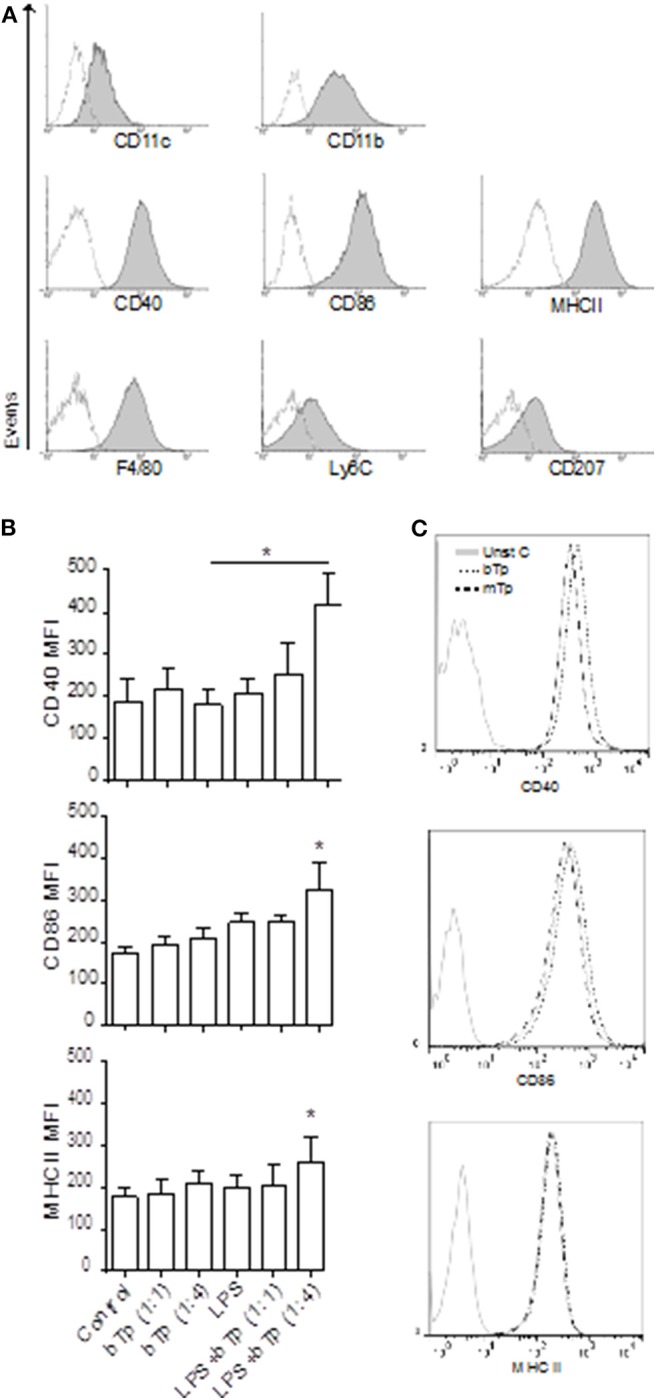
Surface markers in XS106 cell line and comparative expression of activation markers in XS106 stimulated with bTp ± LPS or mTp. **(A)** XS106 were cultured at 1 × 10^6^/well, stained with anti-mouse CD11c, CD11b, CD40, CD86, MHCII, F4/80, Ly6C, CD207 monoclonal Abs and analyzed by flow cytometry. One independent experiment of five is represented by histograms. Dotted line represents negative control for the staining. **(B,C)** 5 × 10^5^ cells XS106 were incubated for 24 h with medium (control), bTp with or without LPS **(B)**, and bTp vs. mTp **(C)**. CD40, CD86, and MHCII were analyzed by flow cytometry. Bar graphic represents MFI (mean fluorescence intensity), mean ± SEM (*n* = 5), *p < 0. 05. One-way ANOVA and Bonferroni's *post-hoc* correction.

As previously reported by Mohan et al. (2005), the XS106 cell line presented surface expression of CD11b and CD11c integrin in accordance with DCs of myeloid origin. In contrast to BMDCs that, in steady state, display low expression of activation markers (Poncini et al., 2008), XS106 cells showed high CD40, CD86, and MHCII surface expression. We also detected the macrophage related marker F4/80 and low expression of the monocytic markers Ly6C and CD207 in XS106, resembling Langerhans cells or migratory DCs in the skin (Figure 1A).

Previous studies have demonstrated that at certain conditions, bTp alone does not affect the activation of BMDCs, measured as MHCII, CD40, CD80, and CD86 surface expression (Poncini et al., 2008). Analyzing here the stimulatory effect of LPS, bTp, or mTp in the XS106 cell line, we found that while these cells displayed a higher basal activation status than BMDCs, neither LPS nor the parasite alone (from 1:1 to 1:10 cell-to-Tp relation, data not shown) enhanced the expression of CD40, CD86, or MHCII. However, LPS plus bTp starting at a 1:4 cell-to-bTp ratio increased the expression of MHCII and the costimulatory molecules CD40 and CD86 (Figure 1B).

The parasite itself, bTp or mTp alone, did not modify the activation status of XS106 in culture (Figure 1C). These results were also observed at 1:10 XS106 cell–Tp relation (data not shown). All data suggest that while bTp can polarize the activation of steady-state BMDCs (Poncini et al., 2008), the parasite could not modify the status of mature DCs such as XS106.

### Comparative Production of TNF and IL-10 in BMDC and XS106 Stimulated With *T. cruzi* Infective Forms

Considering the basal activation of XS106, next we analyzed the production of TNF-α and IL-10, two cytokines with antagonistic properties in culture supernatants. XS106 and BMDCs were stimulated *in vitro* as described in *Materials and Methods*, and then, TNF-α and IL-10 were measured in culture supernatants as previously described by ELISA (Poncini et al., 2008).

**Figure 2:**
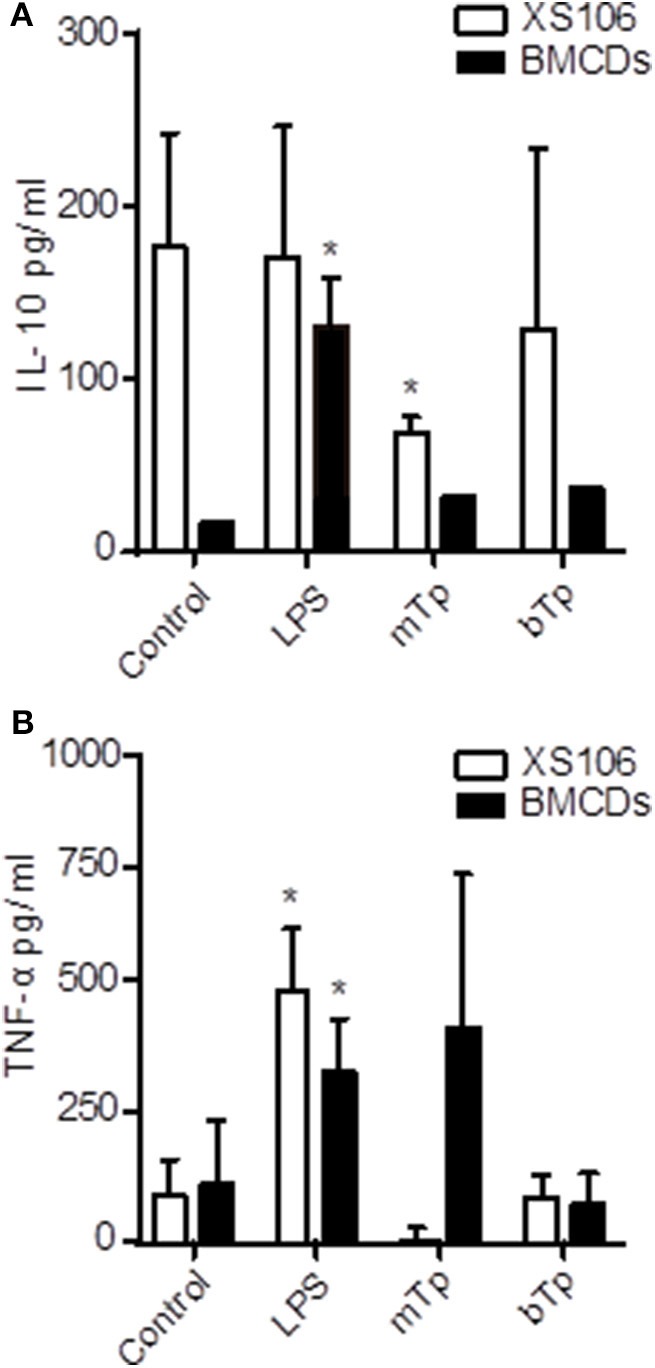
Different IL-10 and TNF-α production patterns in XS106 cell line and BMDCs. 5 x 10^5^ XS106 cells or BMDCs were incubated for 24 h in 10-IMDM (control), LPS, bTp or mTp at a parasite to cell ratio of 4. IL-10 **(A)** and TNF-α **(B)** were measured in culture supernatants by ELISA. ND, not detected. The results are presented as the mean ± SEM of seven independent experiments, *p < 0.05 (compared to the respective control). One-way ANOVA and Dunnett's post Test.

XS106 showed a basal production of IL-10, which was downregulated in the presence of the parasite. The decline in the IL-10 production in XS106 was significant for mTp. As expected for BMDCs, only LPS increased IL-10 secretion (Figure 2A). TNF-α was upregulated by LPS but not by Tp in XS106. In contrast, in BMDCs, both LPS and mTp enlarged the production of this cytokine (Figure 2B), but it was significant only for LPS, results consistent with cellular activation.

### *T. cruzi* Infectivity in BMDC vs. XS106 Cell Line

**Figure 3:**
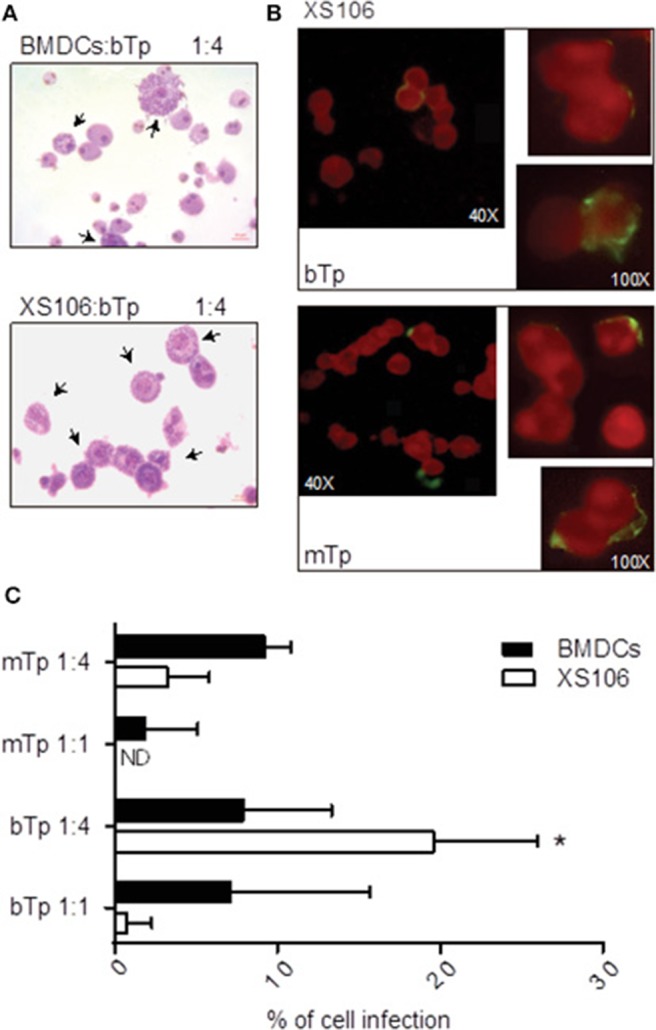
XS106 cell line and BMDCs were differently infected by bTp and mTp. XS106 cells and BMDCs were cultured with bTp or mTp at different cell-to-Tp ratios (1:1 or 1:4). Cell infection was analyzed by optic microscopy after Giemsa staining, at 8 days post-infection **(A)**, 5 days post-infection **(C)**, or by fluorescence microscopy in XS106 cells **(B)** at 5 days post-infection, as described in materials and methods. Mean ± SEM (*n* = 3, 15 random fields counted per treatment). *p < 0.05; unpaired Student's *t*-test. Arrows shown cells with intracellular amastigotes. Bar size: 10 μm **(A)**.

To characterize the invasion and multiplication of the infective stages of *T. cruzi* bTp and mTp in XS106 cells and BMDCs, the parasites were cocultured with cells as described in *Materials and Methods*. Infection was determined and quantified by optic or fluorescence microscopy. Both XS106 cells and BMDCs presented intracellular amastigotes, confirming successful infection by bTp at 8 days post-infection (Figure 3A). However, XS106 cells were two times more infected with bTp than with mTp at the highest ratio of parasites (Figures 3B,C), and in comparison with BMDCs (Figure 3C). In BMDCs, no significant difference was detected in the percentage of infection between bTp and mTp (Figure 3C). These results suggest that at a high ratio of parasite to cell, bTp found a more conducive niche for multiplication in the XS106 cell line than in BMDCs.

### Effect of *T. cruzi* Infective Forms on BMDC and XS106 Capacity to Induce Lymphoproliferation

Previous studies demonstrated that acute infection affects the maturation state of APCs and impairs the T-cell stimulatory capacity of splenic DCs (Alba Soto et al., 2003). In addition, *in vitro* and *in vivo*, we have demonstrated the ability of *T. cruzi* to modulate the differentiation of tolerogenic DCs (Poncini et al., 2008, 2015). *In vitro*, bTps fail to activate BMDCs, prevent the full activation by LPS, and induce high IL-10 secretion and a poor alloresponse (Poncini et al., 2008). Here we comparatively studied the antigen-presenting capacity of both XS106 cells and BMDCs after the stimulation with bTp, mTp, and LPS (positive control) in a model of alloresponse in an MLR.

**Figure 4:**
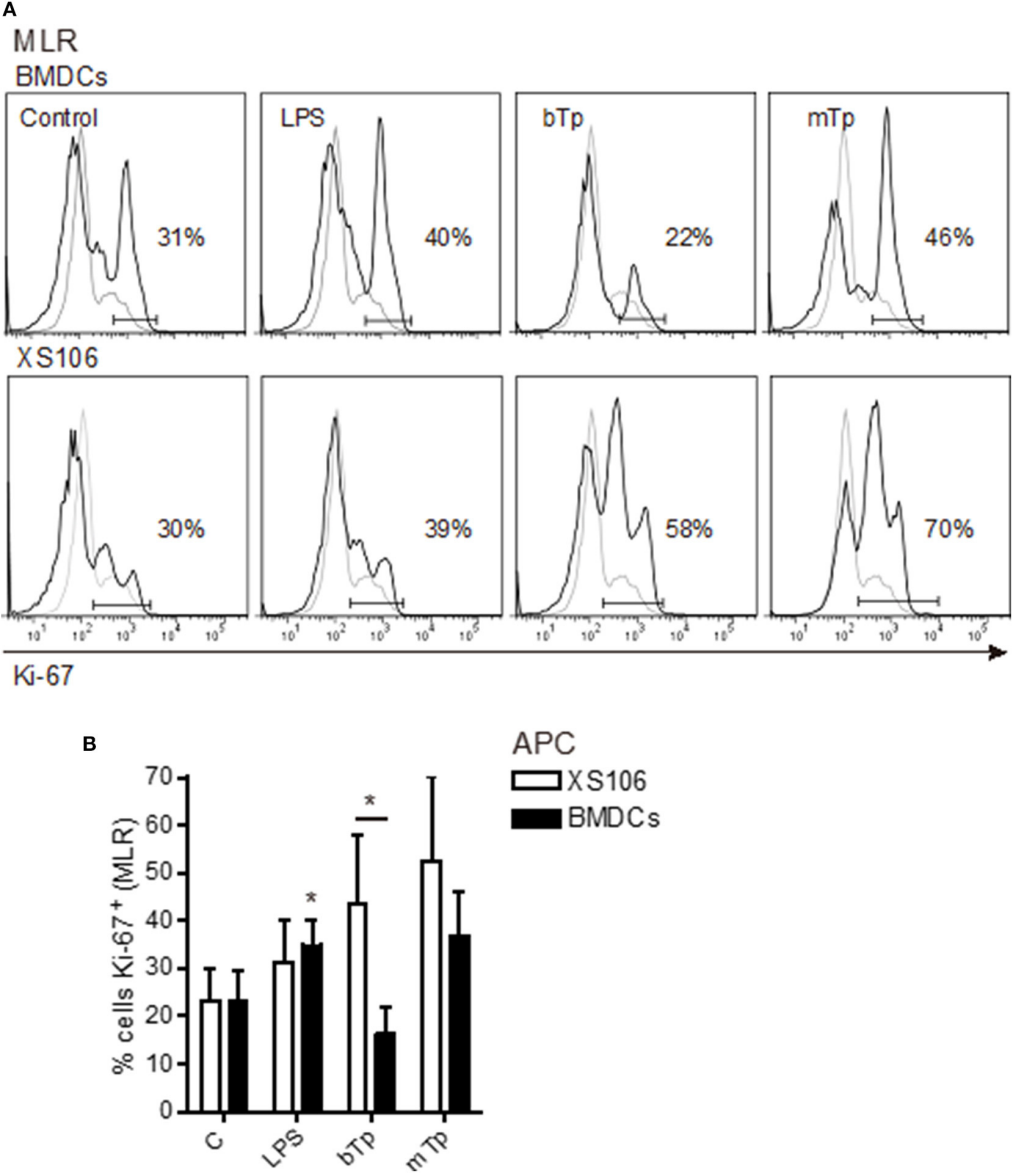
T cell proliferation stimulated by BMDCs or XS106 cells conditioned by different infective stages of *T. cruzi*. BMDCs or XS106 cells were cultured with medium or different stimuli for 24 h. Then, cells were washed, irradiated and cocultured with LN cells enriched in T-cells at 1:10 APCs:LN cells ratio for 3 days. Cell proliferation was assessed by intracellular detection of Ki-67 in T cells obtained from LNs by FACS. One independent experiment of three is represented by histograms. Gray lines show negative controls for the staining, while black lines correspond to cells stained with specific Ab **(A)**. Cultures were set up in triplicate, and results are expressed as the mean ± SEM **(B)**. *p < 0.05; two-way ANOVA and Bonferroni's *post-hoc* correction.

As expected, LPS stimulated the antigen-presenting capacity of BMDCs. Despite not being significant, BMDCs cocultured with bTp presented less capacity to induce lymphoproliferation than mTp-treated BMDCs, which enhanced BMDCs' lymphoproliferative properties (Figures 4A,B). Interestingly, XS106 cells treated with bTp or mTp displayed enhanced capacity to induce T-cell proliferation compared with controls (Figures 4A,B). In addition, bTp-treated XS106 showed better antigen presentation than BMDCs (Figure 4B).

All these results demonstrate that the interaction of different *T. cruzi* infective stages with diverse APCs, but not the cell invasion and the parasite multiplication, could condition the antigen-presenting capacity of different DCs subsets early.

## Discussion

The aim of this work was to characterize the effect of two infective stages of *T. cruzi* on APCs, particularly DCs from a different origin. Previously, our group has demonstrated that bTps promote tolerogenic BMDCs *in vitro* (Poncini et al., 2008). Here, we comparatively studied the interaction of mTp, the representative stage involved in vectorial transmission via the skin or mucous membranes (and gastric epithelium), and bTp responsible for transfusion and congenital transmission with two populations of DCs. The interaction was analyzed *in vitro* by using two different subsets of APCs that could interact with each of the stages described at different biological barriers: on one hand, XS106, a cell line obtained from skin epithelial DCs that resembles LCs (Mohan et al., 2005), and on the other, BMDCs, related to monocyte-derived (Mo)-DCs (Lutz et al., 2016).

The interest in XS106 cell–parasite interaction arises since they are similar to some of the APCs present in the skin. In fact, they presented intermediate expression of CD207 and F4/80 markers, commonly displayed in LCs, several migrating DCs, and some cells from the macrophage linage (Otsuka et al., 2018). In contrast to BMDCs, XS106 displayed a mature/activated phenotype. That difference opened the possibility of exploring the effect of the parasite in DCs at different differentiation/activation statuses, showing that the XS106 cell line and BMDCs responded differently to *T. cruzi*.

mTps are transiently involved in the infection during vectorial transmission. Infection occurs via the skin, and after mTp entry into the host, the parasite suffers intracellular multiplication, as amastigotes and then bTp are present at the site of infection, interacting with resident APCs from the skin or migratory APCs. During this process, the parasite probably conditions the response triggered at the very beginning of the infection at the portal of entry. However, there are no reports yet showing skin APCs as a niche for parasite multiplication. Extended literature describes the wide range of tissues where *T. cruzi* resides in the host (Melo and Brener, 1978; Silva Pereira et al., 2019). Interestingly, a previous study in humans has demonstrated that the parasite can persist in the skin, giving cutaneous lesions associated with the presence of multiplicative amastigotes (Hemmige et al., 2012).

In relation to APCs, it was previously demonstrated in humans that *T. cruzi* modulates the activation state of Mo-DCs *in vitro* (Van Overtvelt et al., 1999) and also during experimental infection (Chaussabel et al., 2003). Our results in the XS106 cell model propose that the basal activation phenotype displayed by mature DCs is not negatively modified by the interaction with the parasite, either mTp nor bTp. Basal IL-10 production was particularly reduced by mTp concomitantly with an enhanced antigen presentation activity, suggesting that mature epidermal DCs conserved their functional properties while interacting with the parasite (both bTp and mTp). In BMDCs, no important changes were detected in comparison to control cells, as previously reported for bTp (Poncini et al., 2008, 2010). However, we found that mTp increased TNF-α production and also antigen presentation in these cells. All results together suggest that *T. cruzi*, depending on the infective stage and the activation/maturation status of the APC, could polarize the immune response during the encounter.

Interestingly, invasion and multiplication of the parasite changed depending on the number of parasites added to cell cultures, the infective stage, and the DCs as recipient cell, demonstrating the importance of the niche the parasite finds to settle up and disseminate during infection. However, Tp invasion and the possibility of the parasite multiplying inside the cell would not be conditioning the antigen-presenting capacity in these two types of DCs, since mTp enhanced the lymphoproliferative capacity of both BMDCs and XS106 cells independently of the percentage of cell infection.

Previous reports with mTp obtained from culture or the vector and bTp from mouse infection showed differences in the immune response associated with the stage of the parasite involved. In a dog experimental model, animals presented enhanced cardiac parasitism and inflammatory response when infected with bTp, suggesting that the inoculum source affects the immunopathological aspects of Chagas disease (de Souza et al., 2014). In addition, it was also demonstrated in a mouse model of infection that bTps were more virulent than culture mTps, via both oral and peritoneal inoculation (Dias et al., 2013), concluding and remarking on the importance of the stage used, its origin, and the model of the infection in order to study the immune response.

Unpublished results from our group demonstrate that the infection with mTp displays reduced cell infiltrate and response at the site of infection (Gutierrez, manuscript under redaction) in comparison with the response described for bTp (Poncini and González-Cappa, 2017). Here, we studied functional properties of BMDCs and XS106 cells stimulated with mTp or bTp in an MLR and found that mTp improved the capacity to induce lymphoproliferation in both cells. In addition, we found that bTp did not impair the antigen-presenting property of XS106 cells, as reported for BMDCs (Poncini et al., 2008), and also that mTps enhance the immunogenicity of BMDCs. These data suggest that infective *T. cruzi* stages could interact and induce diverse effects in different APCs. One interesting conclusion emerging from these results is that apparently, mTp, in contrast to bTp, does not exert tolerogenic signals on DCs. In addition, the tolerogenic properties of bTp seem to be effective only in early differentiated DCs (BMDCs) resembling Mo-DCs in experimental models (Lutz et al., 2016). Those differences between bTp and mTp propose that parasite stages associated to the type of transmission could condition the early immune response and, in part, the outcome of the infection. When the vectorial transmission occurs, the first interaction of the innate immune system with the parasite is with mTp, a stage that, according to our results, would not affect APC immunogenicity. In contrast, when bTps are responsible for the infection (blood transfusion or congenital transmission), its interaction with steady-state DCs or Mo-DCs (in general, monocytes reach inflammatory foci, Poncini and González-Cappa, 2017) could probably impair antigen presentation by these cells, and this could make the immunity against the parasite not strong enough to control *T. cruzi* dissemination.

The importance of the study of *T. cruzi*–APCs interactions opens knowledge to better understand the strategies the parasite has developed in order to evade immunity. In addition, these are key topics on the road to developing new strategies for immunotherapy.

## Data Availability Statement

The datasets generated for this study are available on request to the corresponding author.

## Ethics Statement

The animal study was reviewed and approved by CD N° 04/2015 approved by the University of Buenos Aires's Institutional Committee for the Care and Use of Laboratory Animals (CICUAL).

## Author Contributions

BG, MR, SG-C, and CP contributed the conception and design of the study. BG and CP performed the experiments and analyzed data. EL conducted the epimastigote cultures. BG and CP wrote sections of the manuscript. All authors contributed to manuscript revision and read and approved the submitted version.

### Conflict of Interest

The authors declare that the research was conducted in the absence of any commercial or financial relationships that could be construed as a potential conflict of interest.
